# The effects of low calorie, high protein diet on body composition, duration and sleep quality on obese adults: A randomized clinical trial

**DOI:** 10.1002/hsr2.1699

**Published:** 2023-11-16

**Authors:** Fatemeh Sadat Hashemi Javaheri, A. R. Ostadrahimi, Mohsen Nematy, Seyyed Mostafa Arabi, Mahnaz Amini

**Affiliations:** ^1^ Department of Clinical Nutrition School of Nutrition and Food Sciences Tabriz University of Medical Sciences Tabriz Iran; ^2^ Nutrition Research Center Tabriz University of Medical Sciences Tabriz Iran; ^3^ Department of Nutrition Mashhad University of Medical Sciences Mashhad Iran; ^4^ Department of Basic Sciences, School of Medicine Neyshabur University of Medical Sciences Neyshabur Iran; ^5^ Department of Nutrition, Metabolic Syndrome Research Center Mashhad University of Medical Sciences Mashhad Iran; ^6^ Division of Sleep Medicine, Psychiatry and Behavioral Sciences Research Center Mashhad University of Medical Sciences Mashhad Iran

**Keywords:** apnea, high‐protein diet, low‐calorie diet, obesity, sleep quality

## Abstract

**Background and Aims:**

The effects of high‐protein diets on regulating sleep have received research attention in recent decades. However, no studies have examined the effects of these diets in obese adults. Therefore, this study was conducted to investigate the effects of low‐calorie high protein diets on sleep quality in obese adults.

**Methods:**

This study is a randomized clinical trial conducted on 60 obese adults (BMI > 29.9 kg/m^2^) diagnosed with low‐quality sleep. All participants were given a diet with a 750‐calorie energy deficit. While the control group was given a normal diet, the intervention group received a diet with 30% more protein.

**Results:**

The results showed a significant difference between the control group and intervention group with respect to sleep apnea at 30‐, 60‐, and 90‐day follow‐up (*p* < 0.01). Sleep quality, apnea‐hypopnea index (AHI), sleep latency (SL), and polysomnography were significantly different between the two groups (*p* < 0.05), showing an improvement in sleep quality and obstructive sleep apnea in the intervention group (*p* < 0.05).

**Conclusion:**

This study shows that low‐calorie high‐protein diets can effectively improve apnea, sleep quality, and body composition indices in obese adults.

## 1 INTRODUCTION

Lower sleep quality is an overlooked issue in obese individuals. Sleep serves an essential function and can affect energy maintenance, external appearance, physical health, and body rhythm.^1^, ^2^, ^3^ Evidence shows that sleep deprivation can adversely affect alertness, metabolism, immune system, cortisol and insulin levels, and increase morbidity and mortality.^4^

Chronic sleep disorders affect 10% of the population, while 30% experience sleep disorders some time in their lives.^5^ There is a mutual relationship between obesity and sleep disorders since sleep deprivation makes overeating more likely and therefore causes obesity. People receiving less than 7 h of sleep per day have a higher risk of becoming overweight or obese.^6^ At the same time, obesity increases the risk of chronic diseases such as diabetes, heart disease, stroke, musculoskeletal disorders such as arthritis, and some forms of cancer.^7^, ^8^, ^9^ Therefore, sleep is considered a key factor in psychological and physical health given its profound effects on the immune system, memory, metabolism, and the endocrine system.^10^, ^11^

Diet and micronutrient intake are among the environmental factors influencing sleep quality.^12^, ^13^, ^14^ Although the role of high‐calorie and high‐fat diets in decreasing sleep duration has been shown, it is unclear how a high‐protein diet can affect sleep quality in obese adults.^15^ The results of previous interventional studies show a decrease in sleep latency in response to high‐carbohydrate and high‐fat diets and an increase in sleep quality in response to high‐protein diets (30%–35% protein instead of 10%).^13^, ^14^ Moreover, high‐protein diets improve lipid profile and anthropometric indices, which could contribute to sleep quality in obese adults.^16^ Thus, this study was conducted to examine the effects of a low‐calorie high‐protein diet on sleep quality in obese adults.

## 2 METHODS

This randomized clinical trial was designed according to the Consolidated Standards of Reporting Trials (CONSORT)^3^, ^17^ and Guidelines for Reporting of Statistics for Clinical Research in Urology.^18^ The participants included obese adults (BMI > 29.9 kg/m^2^) who visited the nutrition clinic at Ghaem Hospital (a large research hospital in Mashhad, Iran) to receive a consultation and plan their diets (code of ethics: IR.TBZMED.REC.1401.688). After obtaining informed consent, all healthy individuals meeting the following criteria were enrolled in the study: aged 18–65, no past medical history, obesity diagnosis by a nutritionist, low‐quality sleep diagnosis (PSQI score ≥6 and STOP‐Bang score ≥3). The following additional criteria were also used to screen the participants: no dietary interventions in the past 3 months, no sudden weight loss, and not taking nutritional supplements or herbal medications. The following criteria were used to exclude participants: heart disease, low blood pressure, diabetes, cancer, autoimmune disorders, psychological disorders, overweight, pregnancy, breastfeeding, use of medications that affect sleep quality, and undergoing interventions affecting sleep apnea during the follow‐up.

**Figure 1:**
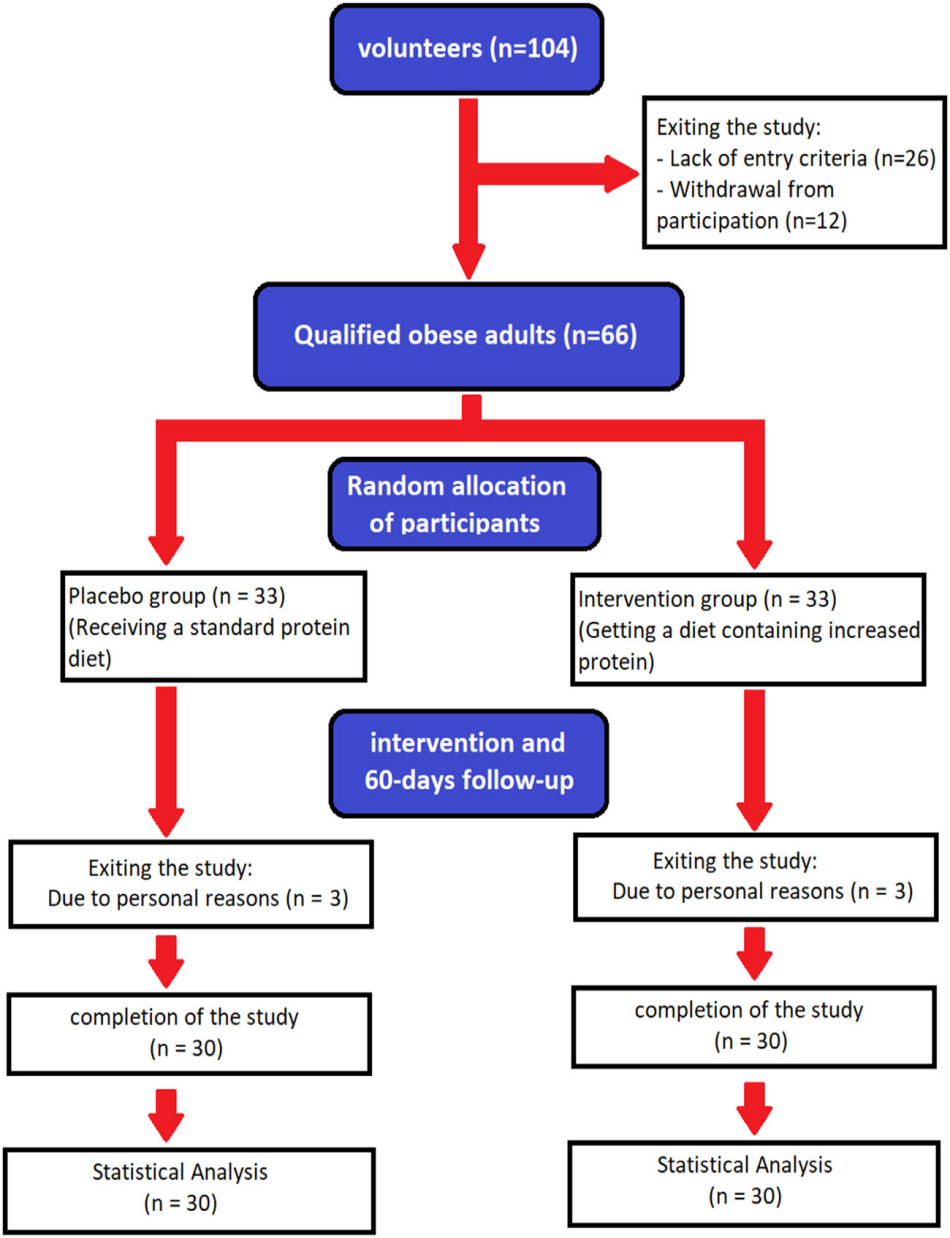
Flowchart of the study.

After entering the study, participants were divided into intervention (low‐calorie high protein diets) and control (low‐calorie with standard amount of protein) groups using block classification based on body mass index (BMI) using sealed enveloped randomization (Figure 1). Randomization and block classification were done using the SEALEDENVELOPE website by a colleague who was not involved in the clinical trial. Demographic, dietary, and anthropometric data were collected following assignment to groups. Height was measured using a stadiometer, weight was measured using a digital scale, BMI was calculated by dividing weight (kg) by height squared (m^2^), and waist circumference was measured with a tape measure in cm. Body composition was evaluated using a BC‐418 segmental body composition analyzer, including fat mass, free fat mass, body water, and muscle mass, as well as the distribution and proportion of each parameter. Daily calorie requirement for each participant was calculated using the Mifflin‐St Jeor equation utilizing age, gender, height, and weight.

Both groups received diets with a 750 calorie energy deficit but the diet for the intervention group contained a larger protein ratio (30% protein compared to 10% in the normal diet). To overcome the calorie restriction and achieve the desired protein percentage, 60% of the protein intake was supplied from high biological value (HBV) sources, and the remaining 40% was supplied using plant‐based sources. HBV sources included red meat (beef and lamb), fish (salmon), eggs, chicken, cheese, and yogurt. Plant sources included peas, lentils, beans, and wheat. To evaluate adherence to the diet, the food frequency questionnaire (FFQ) was completed individually. Diets were analyzed using the N4 software to check calorie intake and micro‐ and macro‐nutrients levels.

Participants' mental health was evaluated using the DASS‐21 Questionnaire (the depression, anxiety, and stress scale–21 items). The reliability and validity of the questionnaire has been shown in several studies. Participants exhibiting any psychological disorders were excluded from the study. Presence of sleep apnea and its severity were assessed using the 8‐item STOP‐Bang questionnaire which takes the following items into account: snoring, daytime sleepiness, obstructive sleep apnea via an observer, high blood pressure, BMI ≥35, age >50, being male, below‐thyroid neck circumference >40 cm. A score of ≥3 on the STOP‐Bang questionnaire indicates the risk of obstructive apnea. The Pittsburgh Sleep Quality Index questionnaire (PSQI) was used to determine sleep quality. An overall PSQI score ≥6 indicates low sleep quality. Finally, the International Physical Activity Questionnaire (IPAQ) was used to evaluate physical activity.

A STOP‐Bang score >5 is suggestive of further sleep apnea disorders. Of the participants meeting this threshold, five were randomly chosen to undergo polysomnography on days 0 and 30 to evaluate apnea‐hypopnea index (AHI), respiratory disturbance index (RDI), oxygen desaturation index, arousal or microarousal index, sleep efficiency, sleep latency, and REM latency. Body composition, anthropometric parameters, and sleep quality were measured on Days 0, 15, 30, 60, and 90 for all participants.

### 2.1 Statistical methods

Descriptive statistics including mean and standard deviation were used to describe the data. The Mann–Whitney test was used to compare the means of nonparametric quantitative variables, and the Friedman test was used to compare the means of nonparametric dependent variables 0, 15, 30, 60, and 90 days after the intervention. Wilcoxon signed‐rank test was used to investigate the difference between the means of dependent nonparametric variables before and after the intervention. Fisher's exact test was used to study the differences between the frequencies of qualitative variables. The statistical analyses were done using Stata v12 (Corp) and *α* ≤ 0.05 was considered statistically significant.

## 3 RESULTS

Overall, 60 people participated in the study in two groups of 30 (intervention and control). The results showed no significant differences in age, gender, anthropometric indices (weight, height, and BMI), body composition (body fat percentage, lean body mass, body water percentage, and muscle mass), and physical activity between the intervention and control groups before the intervention (*p* > 0.05) (Table 1). The means of anthropometric indices were significantly different between the two groups relative to the time of evaluation (*p* < 0.05). Also, mean muscle mass index had increased significantly in the intervention group by the end of the study (*p* < 0.01). Other indices similarly show significant changes over time (*p* < 0.05), except for body water.

**Table 1:** Basic characteristics of participants in the intervention and control groups.

Variables		Intervention (*n* = 30)	Control (*n* = 30)	*p* Value
Age (years)		(96/9) 16/34	(95/9) 40/32	487/0
Height (m)		(11/0) 752/1	(09/0) 758/1	815/0
Weight (kg)		(81/12) 014/99	(61/12) 10/99	979/0
BMI (kg/m^2^)		(42/1) 16/32	(40/1) 93/31	408/0
Waist circumference (cm)		(99/4) 81/103	(08/6) 43/102	678/0
Fat mass index (kg/m^2^)		(28/2) 96/6	(55/1) 35/6	348/0
Fat free mass index (kg/m^2^)		(63/1) 64/7	(39/1) 93/7	329/0
Body water (%)		(81/4) 90/53	(98/4) 00/54	118/0
Muscle mass (kg)		(75/5) 53/66	(87/5) 46/67	750/0
Gender	Female	(67/46) 14	(66/56) 17	606/0
	Male	(33/53) 16	(34/43) 13	
Physical activity	Low	(66/36) 11	(34/33) 10	126/0
	Medium	(34/23) 7	(67/46) 14	
	Severe	12 (40)	(99/19) 6	

There were no significant differences between the two groups 0 and 15 days after the intervention (*p* > 0.05). On the other hand, at 30, 60, and 90 days after the intervention, a significant difference was observed between the mean sleep apnea scores of the two groups (*p* < 0.01) showing an improvement in sleep apnea in the intervention group (*p* < 0.01). The control group showed no significant change in sleep apnea score over the course of the study (*p* > 0.05) (Table 2).

**Table 2:** Sleep quality and sleep apnea scores for the intervention and control groups.

Sleep assessment		Intervention (*n* = 30)	Control (*n* = 30)	*p* Value
STOP‐Bang Score	Before	(55/1) 16/4	(41/1) 73/4	202/0
	Day 15	(15/1) 71/3	(08/1) 30/4	06/0
	Day 30	(02/1) 26/3	(15/1) 34/4	001/0
	Day 60	(25/1) 73/2	(19/1) 13/4	001/0
	Day 90	(718/0) 36/2	(01/1) 93/3	001/0
	*p* Value^b^	001/0	115/0	
PSQI Score	Before	(59/2) 67/7	(57/2) 30/8	355/0
	Day 15	(01/2) 16/6	(11/2) 93/6	139/0
	Day 30	(78/1) 70/4	(23/2) 80/7	001/0
	Day 60	(56/1) 43/4	(99/1) 37/7	001/0
	Day 90	(66/1) 84/3	(24/2) 73/7	001/0
	*p* Value^b^	001/0	179/0	

b Friedman test.

**Figure 2:**
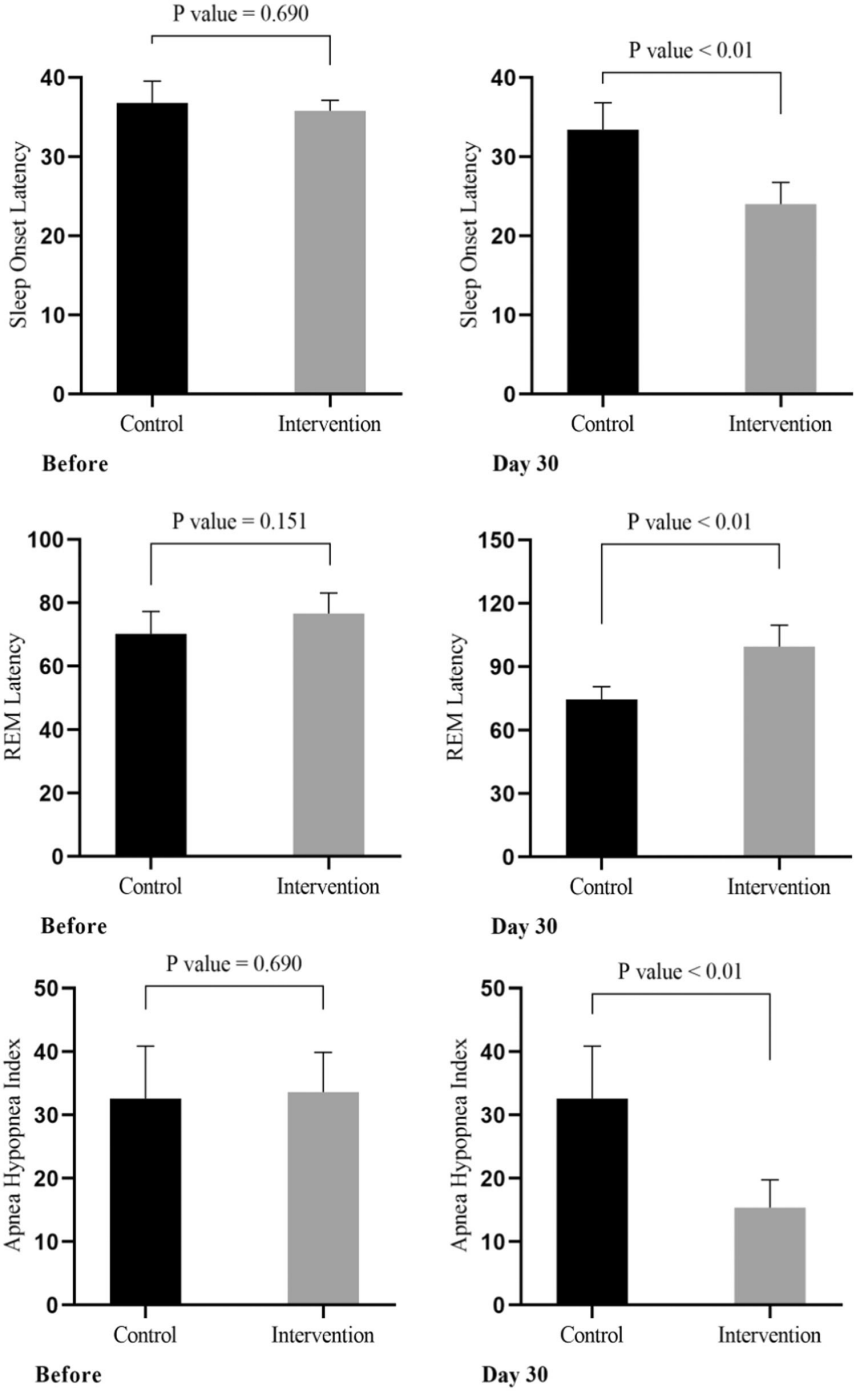
Mean polysomnography scores for the intervention and control groups, before and 30 days after the intervention.

There were no significant differences between the two groups in terms of average sleep quality at Days 0 and 15 (*p* > 0.05). However, the average scores had changed significantly by days 30, 60, and 90 (*p* < 0.01) (Table 2). The polysomnography test scores showed no significant difference at Day 0 (*p* > 0.05), while a significant difference emerged between the two groups 30 days after the intervention, with the intervention group showing an improvement in their test results (*p* < 0.05). Polysomnographic indices were significantly different between the two groups after the intervention (*p* < 0.5) (Table 3) (Figure 2).

**Table 3:** Polysomnographic indices for the intervention and control groups.

Polysomnography		Intervention (*n* = 30)	Control (*n* = 30)	*p* Value
Sleep onset latency (min)	Before	(58/0) 83/35	(24/1) 81/36	690/0
	Day 30	(22/1) 00/24	(53/1) 40/33	002/0
	*p* Value	043/0	131/0	
REM latncy (min)	Before	(92/2) 6/76	(13/3) 2/70	151/0
	Day 30	(54/4) 4/99	(73/2) 4/74	01/0
	*p* Value	01/0	225/0	
TST (min)	Before	(13/16) 6/603	(02/13) 8/597	995/0
	Day 30	(80/21) 0/529	(19/21) 1/625	016/0
	*p* Value	01/0	500/0	
Oxygen desaturation index (per hour)	Before	(34/1) 0/16	(21/1) 40/15	690/0
	Day 30	(11/1) 80/5	(35/1) 2/14	01/0
	*p* Value	01/0	498/0	
T90	Before	(41/3) 2/67	(49/2) 2/63	421/0
	Day 30	(22/2) 4/46	(80/1) 6/64	01/0
	*p* Value	01/0	587/0	
AHI (per hour)	Before	(80/2) 6/33	(93/3) 4/31	690/0
	Day 30	(93/1) 4/15	(71/3) 6/32	001/0
	*p* Value	01/0	680/0	
RDI (per hour)	Before	(62/4) 6/32	(91/2) 0/33	990/0
	Day 30	(55/3) 6/13	(84/3) 1/37	008/0
	*p* Value	01/0	102/0	
AHI in REM (per hour)	Before	(01/2) 4/41	(30/3) 8/34	222/0
	Day 30	(90/2) 8/13	(96/3) 2/33	01/0
	*p* Value	01/0	786/0	
Snoringindex (per hour)	Before	(21/4) 12/37	(82/4) 41/39	547/0
	Day 30	(71/6) 18/7	(27/6) 57/27	01/0
	*p* Value	01/0	04/0	
Snoring index in REM (per hour)	Before	(44/5) 71/28	(49/6) 76/31	841/0
	Day 30	(84/6) 31/11	(14/7) 47/25	01/0
	*p* Value	01/0	471/0	

## 4 DISCUSSION

This study aimed to investigate the effects of low‐calorie high‐protein diets on anthropometric indices and sleep quality in obese adults. The results showed that such diets can improve sleep quality and the related indices and enhance anthropometric indices and body composition, which is in line with previous studies in this field.^7^, ^8^, ^9^ Diet can act as a powerful regulating factor by improving anthropometric indices and sleep cycle.^19^ The evidence suggests that certain macro‐ and micronutrients and diets can improve sleep quality and regulate the circadian rhythm.^14^, ^20^ This shows the significant prospect of improving sleep quality through diets and nutrition.

The positive effect of the protein content in a diet and its desirable health outcomes such as weight control and improved body composition have been reported in the literature.^21^, ^22^ Our results similarly showed that obese adults consuming low‐calorie high‐protein diets have significantly better body composition and anthropometric indices compared to the control group when confounding factors such as physical activity are taken into account. In their 12‐week study using a diet with a 750‐Calorie deficit, Hudson and colleagues found that the BMI of all participants decreased.^23^ High‐protein diets decrease weight through a number of mechanisms including enhancing satiety, maintaining metabolism and lean body mass while losing weight, and increasing the thermal effect.^24^, ^25^ Westerterp and colleagues also showed that high‐protein diets (20% protein instead of 10%) can cause a 50% weight loss over 3 months, which can be attributed to improved satiety and decreased energy efficiency.^26^ In this study, fat mass had significantly decreased in the intervention group by the end of the intervention, while muscle mass had notably increased. This can be due to higher protein synthesis, increased protein accumulation in tissues, and inhibition of protein decomposition.^27^, ^28^

Overweight, obesity, and central adipose tissue accumulation are among the risk factors affecting sleep quality. Lifestyle changes resulting in the reduction of adipose tissue and weight loss through a balanced diet are the most important interventions to the mitigate risk. A number of studies have shown that high‐protein diets enhance sleep quality indices,^22^, ^29^, ^30^ although some studies do not report an explicit relationship between high protein intake and sleep quality.^16^ Sutanto and colleagues showed that dietary tryptophan has a significant positive correlation with sleep duration.^31^ However, they reported that that there is no clear correlation between high‐protein diets and sleep quality and latency. Halson and colleagues state that increasing carbohydrate intake can decrease sleep latency and increasing protein intake can increase sleep quality. They also found that some high‐fat diets can cause a decrease in sleep duration,^13^ similar to our results. High protein ratios (35% instead of 10%) lead to an increase in satiety after a meal, while carbohydrates and fat, at least in high quantities, may reduce the satiety quotient.^14^ Furthermore, due to their effects on serotonin and melatonin production and secretion, there is an interest in amino acids, especially tryptophan, as soporific nutrients.^14^

Dietary interventions are one of the priorities in obesity risk management. In long trials, a 500‐calorie deficit has been found to be the best way to lose weight.^32^ Surratt and colleagues showed that very low calorie diets (VLCD) can improve obstructive sleep apnea.^33^ In our study, polysomnography showed that AHI improved significantly in people consuming a low‐calorie high‐protein diet compared with a normal calorie deficient diet. Physical activity can act as a confounding factor for body composition, so its effects were controlled and adjusted in our analyses. Kansanen and colleagues showed that weight loss with VLCD is an effective treatment for obstructive sleep apnea. Additionally, weight loss under VLCD significantly improved sleep apnea and had a positive effect on blood pressure.^34^ Vgontzas and colleagues showed that obese adults without sleep disorders or emotional stress and nonobese controls had similar sleep durations, which highlights the importance of diagnosis and treatment of sleep disorders as a potential treatment for obesity.^35^

Hashimoto and colleagues state that increasing energy intake and diet quality (e.g., increasing mineral and vitamin intake along with tryptophan) improve sleep quality.^36^ Zhou and colleagues showed that weight loss using a high‐protein diet caused an improvement in sleep quality in the intervention group regardless of protein source. The intervention groups consumed diets with 20% or 30% protein while the controls were prescribed a 3‐week balanced diet, containing 0.8 g of protein/kg. These findings are in line with ours, emphasizing the effects of low‐calorie high‐protein diets on sleep quality in obese adults. The intervention group in our study experienced improved sleep quality while the control group did not experience any significant changes.

## 5 CONCLUSION

The results of this study show that low‐calorie high‐protein diets can promote sleep quality in obese adults when controlling for confounding factors such as physical activity. Moreover, the results of polysomnography 30 days after the intervention reveal that sleep quality indices are significantly improved in the intervention group compared with the control group.

## AUTHOR CONTRIBUTIONS

**Fatemeh Sadat Hashemi Javaheri**: Data curation; formal analysis; investigation; methodology; writing—original draft. **A. R. Ostadrahimi**: Conceptualization; methodology; project administration; supervision; validation. **Mohsen Nematy**: Conceptualization; supervision; validation. **Seyyed Mostafa Arabi**: Conceptualization; methodology; writing—review & editing. **Mahnaz Amini**: Investigation; resources; supervision; validation.

## CONFLICT OF INTEREST STATEMENT

The authors declare no conflict of interest.

## TRANSPARENCY STATEMENT

The lead author A. R. Ostadrahimi affirms that this manuscript is an honest, accurate, and transparent account of the study being reported; that no important aspects of the study have been omitted; and that any discrepancies from the study as planned (and, if relevant, registered) have been explained.

## ETHICS STATEMENT

This study was approved by the ethics board of Tabriz University of Medical Sciences under the registration code IR.TBZMED.REC.1401.688.

## Data Availability

The original contributions presented in the study are included in the article/supplementary materials. Further inquiries can be directed to the corresponding author.
